# Prevalence and Risk Factors for Toxoplasmosis in Middle Java, Indonesia

**DOI:** 10.1007/s10393-016-1198-5

**Published:** 2016-11-09

**Authors:** Annisa Retmanasari, Barandi Sapta Widartono, Mahardika Agus Wijayanti, Wayan Tunas Artama

**Affiliations:** 1 Postgraduate Program of Tropical Medicine, Faculty of Medicine, Universitas Gadjah Mada, Yogyakarta, Indonesia; 2 Departement of GISRP, Faculty of Geography, Universitas Gadjah Mada, Yogyakarta, Indonesia; 3 Faculty of Medicine, Universitas Gadjah Mada, Yogyakarta, Indonesia; 4 Departement of Biochemistry, Medical Veterinary Faculty, Universitas Gadjah Mada, Jl. Fauna 2 Karangmalang, Yogyakarta, 55281 Indonesia; 5 EcoHealth Resource Center (EHRC)-Universitas Gadjah Mada, Yogyakarta, Indonesia

**Keywords:** toxoplasmosis, seroprevalence, risk factors, geographic information system (GIS), EcoHealth

## Abstract

Toxoplasmosis is a zoonosis caused by *Toxoplasma gondii*. Risk factors include consumption of undercooked meat, raw vegetables, and unfiltered water. This study aims to determine the seroprevalence and spatial distribution of toxoplasmosis in Middle Java, Indonesia, using an EcoHealth approach, combined with geographic information system (GIS). A total of 630 participants were randomly selected from seven districts. Each participant completed a questionnaire and provided a blood sample. The seroprevalence of toxoplasmosis was 62.5%. Of those who were seropositive, 90.1% were IgG+, and 9.9% were IgG+ and IgM+. Several risk factors were identified, including living at elevations of ≤200 m, compared with >200 m (OR = 56.2; *P* < 0.001), daily contact with raw meat (OR = 1.8; *P* = 0.001), unfiltered water (OR = 1.7; *P* = 0.003), and density of cats (OR = 1.4; *P* = 0.045). Visualizing the spatial distribution of seropositive respondents highlighted clustering in lowland areas. This study highlighted that Middle Java has a high prevalence of toxoplasmosis and identified some important environmental, ecological, and demographic risk factors. When researching diseases, such as toxoplasmosis, where animal hosts, human lifestyle, and environmental factors are involved in transmission, an EcoHealth method is essential to ensure a fully collaborative approach to developing interventions to reduce the risk of transmission in high-risk populations.

## Introduction and Purpose

Toxoplasmosis, a zoonotic disease prevalent worldwide, is caused by the protozoan parasite *Toxoplasma gondii* (*T. gondii*). The lifecycle of this parasite has three stages: the oocyst, bradyzoite (cyst), and tachyzoite. Tachyzoites actively and rapidly invade hosts cells, aided by one of the most prevalent proteins: Granule-1 (GRA-1). This protein is responsible for binding with the calcium released into the lumen of the vacuole and interacting with tissue (Weis and Kami 1995; Lee et al. 2014). Toxoplasmosis can lead to congenital malformation in the intermediate host, abortion, and hydrocephalus (Fusco et al. 2007; Tavassoli et al. 2013).

Worldwide, over 6 billion people have been infected with toxoplasmosis (Klaren and Kijlsstra 2002). The seroprevalence of toxoplasmosis in humans varies in different countries, with previous studies reporting country-specific prevalence rates of 6.7% in Korea; 12.3% in China; 23.9% in Nigeria; 46.0% in Tanzania, and high prevalence of up to 98.0% in some regions (Glasner et al. 1992; Fromont et al. 2009; Kamani et al. 2009; Shin et al. 2009; Swai and Schoonman 2009; and Xiao et al. 2010). Serological studies in livestock revealed that worldwide the average *T. gondii* prevalence is 14.0% in cattle; 27.0% in goats; 25.0% in pigs; 66.0% in sheep; and 44.0% in horses (Tenter et al. 2000). In Indonesia, the prevalence of toxoplasmosis in livestock has been estimated as 8.8% in cattle, 51.0% in goats, and 45.0% in sheep (Artama et al. 2007). The prevalence of human toxoplasmosis in Indonesia for humans has been reported from 43 to 88% in some regions (Subekti et al. 2006).

Transmission of toxoplasmosis can occur through two mechanisms: vertically from mother to fetus and horizontally through the consumption of undercooked meat, contaminated milk, and raw vegetables, and also in the case of transplants and accidents in laboratory (Fusco et al. 2007; Gangneux and Marie, 2012; Uttah et al. 2013; and Wing 2016). Members of the family *Felidae* (domestic cats) are the definitive hosts; however, many mammals (live stocks and humans), birds, and fishes are intermediate hosts (Gilot-Fromont et al. 2012; Webster et al. 2013). A single cat can shed more than 100 million oocysts in prepatent period about 18 days. In order for these oocysts to become infectious, they must undergo sporulation, which can take more than 4 days and occurs in environments with high humidity and a low temperature. Oocysts can survive outdoors for many months, remain viable for long periods of time in water, and resist freezing and moderately high water temperatures. Tissue cysts in meat are usually destroyed by heating to 67°C. The survival of tissue cysts at lower temperatures depends on the duration of cooking (Tenter et al. 2000).

Country-specific environmental conditions, eating habits, hygiene, and host susceptibility all contribute to global differences in the prevalence of toxoplasmosis (Furtado et al. 2011). Indonesia has a tropical climate, providing suitable conditions for gardening and farming, increasing human contact with soil and livestock, as well as the possibility of increasing contact with raw meat. Additionally, it is common to consume goat, sheep, beef, chicken, and rabbit satay, potentially with undercooked meat and raw vegetables. In a multicenter study in Europe, meat consumption was estimated to be responsible for between 30.0 and 63.0% of toxoplasmosis cases, while soil contact was identified as the cause of between 6.0 and 17.0% of cases (Gangneux and Marie 2012).

Health and disease are increasingly understood as the product of interrelated ecological, cultural, social, and economic situations (Blazkuez et al. 2014). Socio-demographic status, lifestyle, and environment are risk factors for toxoplasmosis; therefore, interdisciplinary collaborative research is essential to reduce the risk of transmission. EcoHealth is one of several integrative approaches that considers interactions between health and the environment, making it an ideal approach for researching a zoonosis such as toxoplasmosis.

Previous research on toxoplasmosis has identified high-risk geographical areas; however, further investigation using spatial techniques will achieve more accurate identification of high-risk locations, which can be used to improve strategies to prevent the spread of this disease. Geographic Information Systems (GIS) have been applied in several situations, including surveillance and monitoring of diseases (Jores et al. 2008), disease cluster detection (Miller et al. 2004), identification of environmental predictors of disease in wildlife populations (Miller et al. 2002), risk assessments (Hung et al. 2004, 2007), and modeling the spread and impact of disease (Norman 2008). In the present study, the use of GIS, combined with a seroprevalence study and risk factor questionnaire, demonstrates a true EcoHealth approach, which is essential when researching diseases, such as toxoplasmosis, where animal hosts, human lifestyle, and environmental factors are all involved in transmission. The use of GIS will aid in the identification of high-risk locations, enabling public health interventions to be targeted at specific populations in Middle Java. This system was constructed utilizing the administrative boundaries (at provincial and municipal levels) of the Campania region with toxoplasmosis cases (Fusco et al. 2007).

Using an EcoHealth approach, this study aims to determine the seroprevalence and spatial distribution of toxoplasmosis in Middle Java, and identify factors associated with the occurrence of toxoplasmosis.

## Methods

### Setting and Design

A cross-sectional study was conducted in 2014 in seven districts in Middle Java that were divided into three clusters: Cluster 1 (Purworejo and Kebumen); Cluster 2 (Cilacap and Banyumas); and Cluster 3 (Purbalingga, Banjarnegara and Wonosobo). The distribution of the sample was determined based on geographical location and topography of the region. The districts were chosen to be representative of the Middle Java region. The required sample size was calculated to be 588 with 10% non-response rate; a rounded-up sample size of 630 respondents recruited. It was calculated using proportional sample size as follows:

$$ {\text{Sample}}\,{\text{size}} = \frac{{Z_{{1 - \alpha /2^{2} }} p(1 - p)}}{{d^{2} }} $$

Here *Z*_1−*α*/2_ is the Standard normal variation [at 5% type error (*P* < 0.05)], *P* is Expected proportion in population based on previous studies or pilot studies, and d is the Absolute error or precision (has to be decided by researcher)

(Charan and Tamoghna 2013)

According to previously published studies, using a toxoplasmosis estimate of 15% and assuming a 95% confidence interval, sampling was performed using a multistage cluster sampling method. In the first stage, the selection of the research clusters (districts) and sub-clusters (subdistrict, village, and subvillage) was performed based on probability proportionate to size (PPS). In the second stage, households were randomly selected in each district using a simple random sampling technique (Rosen 1997; United Nations 2005). Participants were approached by the community leader and research team, and they were asked if they wanted to participate in the study. Informed consent was sought from all participants prior to enrolment in the study. Blood samples were collected from participants by venipuncture using sterile syringes and transferred into serum separator tubes. The tubes were labeled and transported to the laboratory in thermic bags at 2–8°C.

### Survey Design

In addition, a questionnaire was completed by all respondents that included demographics, diet (consumption of undercooked meat, raw vegetables, and raw water), daily activity (interaction with cats, contact with raw meat, and daily contact with soil), and local environment (water resource, temperature, elevation, distance from river, humidity, and population of cats). The cat population in square kilometer was considered to be of lower density if the population was less than 15, and higher density if the population was more than 15.

### Serum Collection and Testing

A sample of up to 3cc of blood was obtained from each participant. Blood samples were stored overnight in the refrigerator at 4°C. The serum was separated from the blood and transferred to eppendorf tubes to measure IgM and IgG. Serum was stored in a freezer at −20°C. Serum samples were screened for *T. gondii* using an indirect ELISA (Rahbari et al. 2012), using the recombinant GRA-1 tachyzoite antigen (Sulistyaningsih et al. 2005).

### Spatial Data Collection

GIS software (ArcGIS 10.1) produced by ESRI was used to map the spatial distribution of *T. gondii*. The administrative map was provided by the Center for Research, Promotion and Cooperation Geospatial Information Agency (http://www.bakosurtanal.go.id/peta-rupabumi/); the watershed map was provided by the Center for Data Processing Ministry of Public Works and Public Housings (http://sigi.pu.go.id/); and the topography map was provided by the United States Geological Survey (.srtm format). The household’s distance from the river was collected from interview data and generated using Google Earth produced by Google. Environmental data, such as temperature, were collected using digital readings in field. Spatial distribution was visualized using overlay (ESRI 2001), which combines digital maps with attribute data.

### Statistical Analysis

A chi-square test (bivariate test) from SPSS Statistic 22 produced by IBM was used to determine factors associated with the seroprevalence of *T. gondii*. Statistical significance was set at a value of *P* < 0.05.

## Results

### Seroprevalence of Toxoplasmosis in Middle Java

All participants were notified prior to the household visit and only one respondent (between the ages of 15–60) per household was interviewed and asked to provide a blood sample. A total of 630 samples were collected from the surveyed households in Middle Java. The results identified the prevalence of toxoplasmosis was (*n* = 394; 62.5%) (Table 1). Thirty-nine respondents (9.9%) were seropositive for IgG and IgM, with the remaining respondents (*n* = 355/394; 90.1%) being seropositive for IgG (Table 2).

**Table 1:** Seroprevalence of Toxoplasmosis Based on IgG and IgM Anti-toxoplasma Examination by ELISA Using GRA-1 Tachyzoite Local Isolate Recombinant Proteins of *T. gondii* in Middle Java, 2014.

Toxoplasmosis	All respondents *n*	Percentage (%)
Positive (*n* = 630)	394	62.54
Negative (*n* = 630)	236	37.46

**Table 2:** Results of IgG and IgM Anti-toxoplasma Examination in Middle Java, 2014.

Category	All respondents *n*	Percentage (%)
IgG (+) and IgM (+) (*n* = 630)	39	9.89
IgG (+) and IgM (−) (*n* = 630)	355	90.10

### Risk Factors for Toxoplasmosis in Middle Java

Several risk factors for toxoplasmosis in Middle Java were identified (Table 3). The greatest risk was presented by an elevation at or under 200 m, compared with an elevation over 200 m (CI = 27.65–114.23; OR = 56.19; *P* < 0.001). Respondents whose daily work or other activities included direct contact with raw meat were at increased risk compared with those who did not have regular contact with raw meat (CI = 1.27–2.47; OR = 1.77; *P* = 0.001). The risk of toxoplasmosis was also higher for respondents who did not filter their water (CI = 1.22–2.49; OR = 1.74; *P* = 0.003), and for those who lived in an area with a high population of cats (CI = 1.02–1.98; OR = 1.42; *P* = 0.045).

**Table 3:** Risk Factors for Toxoplasmosis in a Sample of 630 People in Middle Java, 2014.

Variable	Prevalence *n* (%)	OR (95% CI) (*Bivariate analysis*)	*P* value
Sex		0.90 (0.65–1.27)	0.023*
Women (*n* = 353)	236 (66.85)		
Men (*n* = 247)	158 (63.97)		
Interaction with cats		0.72 (0.50–1.03)	0.092
Yes (*n* = 438)	264 (60.27)		
Never (*n* = 192)	130 (67.71)		
Density of cat		1.42 (1.02–1.98)*	0.045*
High (*n* = 263)	177 (67.30)		
Low (*n* = 367)	217 (59.12)		
Consumption of undercooked meat		0.85 (0.62–1.18)	0.386
Yes (*n* = 329)	200 (60.79)		
Never (*n* = 301)	194 (64.45)		
Consumption of raw vegetables		0.41 (0.09–1.96)	0.412
Yes (*n* = 580)	386 (66.55)		
Never (*n* = 50)	8 (16.00)		
Contact with raw meat		1.77 (1.27–2.46)*	0.001*
Yes (*n* = 385)	261 (67.80)		
No (*n* = 245)	133 (54.28)		
Consumed raw water		0.73 (0.51–1.04)	0.098
Yes (*n* = 180)	103 (57.22)		
No (*n* = 450)	291 (64.67)		
Water resource		1.74 (1.22–2.48)*	0.003*
Without filtration (*n* = 458)	303 (66.16)		
With filtration (*n* = 172)	91 (52.91)		
Daily contact with soil		0.58 (0.30–1.12)	0.136
Yes (*n* = 580)	358 (61.72)		
No (*n* = 50)	36 (72)		
Temperature		0.57 (0.41–0.81)	0.002*
<29°C (*n* = 192)	103 (53.65)		
≥29°C (*n* = 437)	291 (66.59)		
Elevation		56.19 (27.65–114.23)*	0.000*
≤200 m (*n* = 487)	385 (79.05)		
>200 m (*n* = 143)	9 (6.29)		
Distance from river		0.27 (0.13–0.56)	0.000*
≤500 m (*n* = 336)	236 (70.24)		
>500 m (*n* = 52)	42 (80.77)		
Humidity		0.69 (0.36–1.32)	0.330
<60% (*n* = 47)	33 (70.21)		
>60% (*n* = 583)	361 (61.92)		

* Significant with *P* value <0.05.

**Figure 1:**
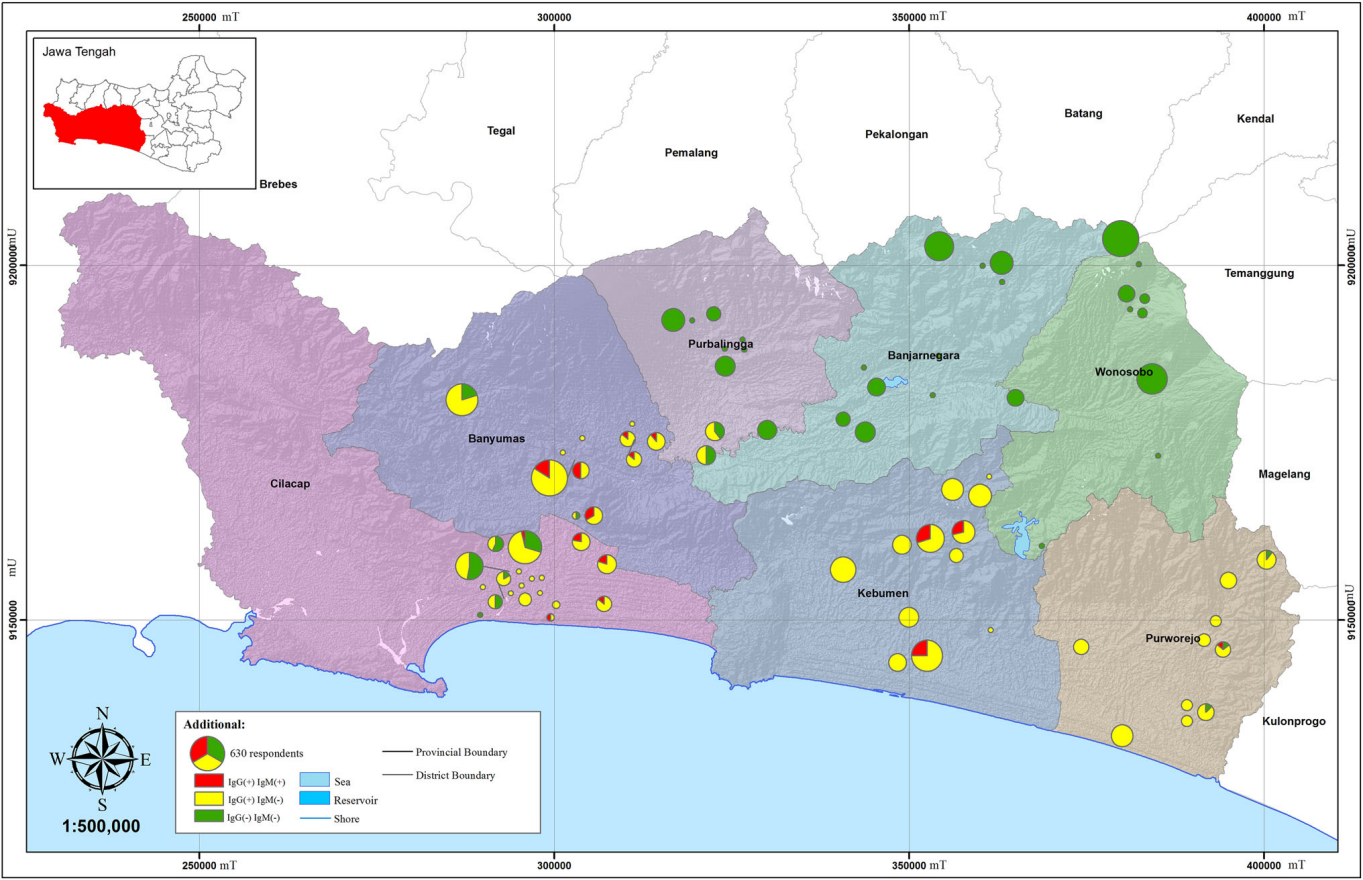
Spatial distribution of toxoplasmosis based on administration boundaries in Middle Java, Indonesia 2014.

**Figure 2:**
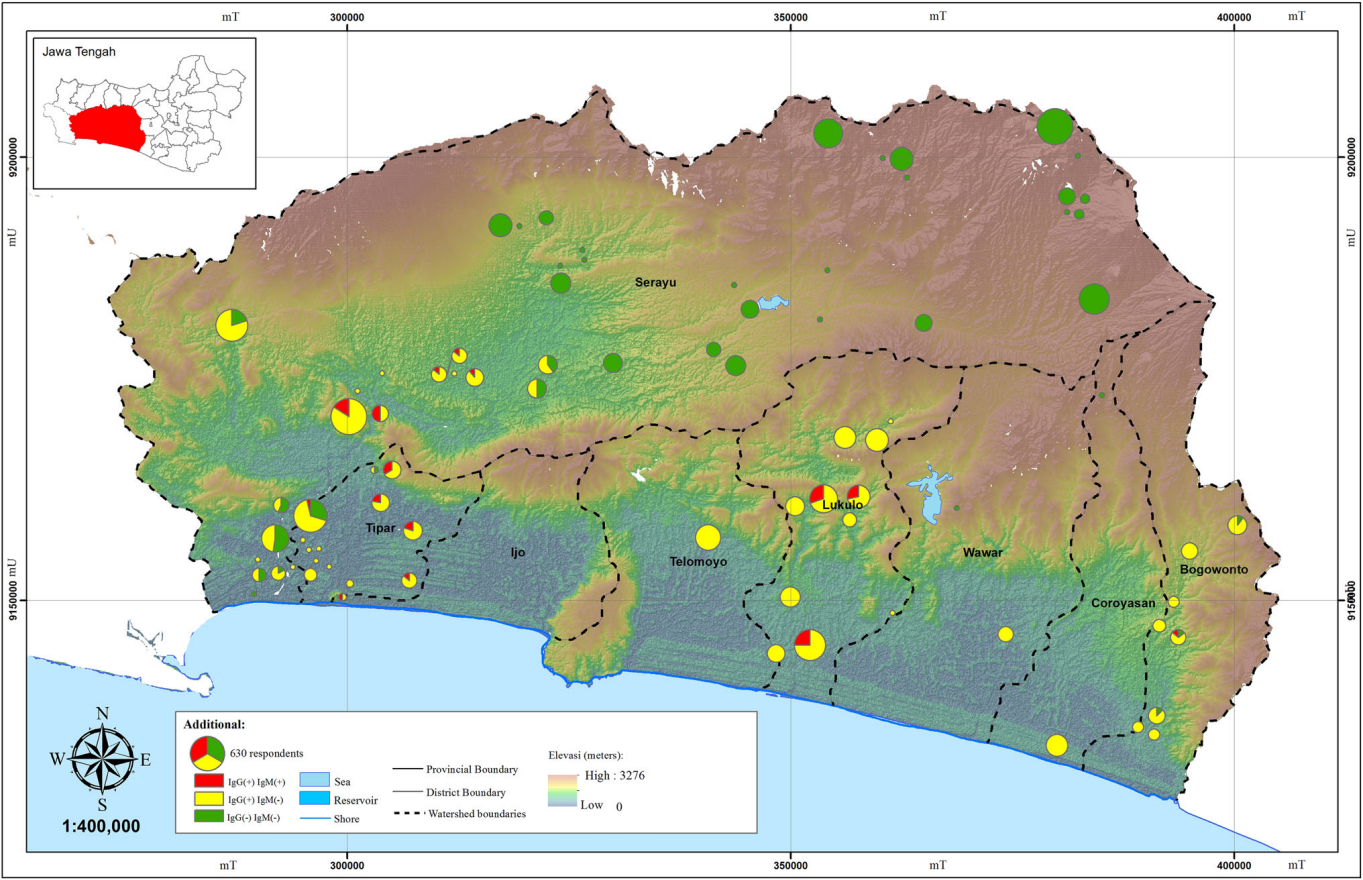
Correlation of toxoplasmosis with elevation on watershed’s Middle Java, Indonesia 2014.

Risk factors for toxoplasmosis varied depending on geographical location; each district presented different risks (Fig. 1; Table 4). The districts at a higher elevation, Wonosobo and Banjarnegara, had no cases of toxoplasmosis (Fig. 2). Purworejo, Kebumen, Banyumas, Purbalingga, Banjarnegara, and Wonosobo districts had the highest proportion of respondents who did not use filtered water, ranging from 91.4% in Kebumen to 55.7% in Purbalingga (Table 4). The highest proportion of respondents who reported the population of cats in their district was high are Purworejo (58.6%) and Kebumen (52.9%). Purworejo, Kebumen, Banyumas, Purbalingga, and Wonosobo districts reported as the largest proportions of respondents with high rates of contact with raw meat during their daily work or other activities, ranging from 75.0% in Kebumen to 57.1% in Purbalingga (Table 4). Additionally, 570 respondents (90.5%) did not use gloves when handling raw meat.

**Table 4:** Percentage of Toxoplasmosis Risk Factors in Each District of Middle Java.

District	Risk factors (%)					
	Water sources		Population of cats		Contact with raw meat	
	Without filtration	With filtration	Many	Few	In work	Not in work
Purworejo	82.86	17.14	58.57	41.43	70.00	30.00
Kebumen	91.43	8.57	52.86	47.14	75.00	25.00
Cilacap	47.72	52.78	29.03	70.37	47.22	52.78
Banyumas	65.69	34.31	31.32	68.63	65.69	34.31
Purbalingga	55.71	44.29	48.57	51.43	57.14	42.86
Banjarnegara	85.74	14.26	34.29	65.71	47.14	52.86
Wonosobo	78.57	21.43	37.14	62.86	57.14	42.86

### Spatial Distribution of Toxoplasmosis in Middle Java

The spatial distribution of toxoplasmosis combined with elevation is illustrated in Fig. 1 and with the watershed in Fig. 2. The lowland districts of Purworejo, Kebumen, and Banyumas had predominantly chronic toxoplasmosis infections, IgG (+) and IgM (−), illustrated in yellow on the map. Recent infection with toxoplasmosis, (IgG (+) and IgM (+), is represented by red (Figs. 1, 2). Cilacap (a lowland district) and Purbalingga (a highland district) had predominantly chronic infections or negative samples, IgG (−) and IgM (−), shown in green. Districts with no positive samples, Banjarnegara and Wonosobo, are located in mountainous or hilly locations, over 500 m.

This study was conducted in the watershed regions of Serayu, Tipar, Lukulo, and Bogowonto (Fig. 2). Tipar, Lukulo, and Bogowonto have a similar seroprevalence of toxoplasmosis. Within the Serayu watershed, there were two distinct groups of cases, with no cases upstream of Serayu (Wonosobo and Banjarnegara districts). Cases of toxoplasmosis were identified in Purbalingga and downstream from the river in Cilacap.

## Discussion

In this study, the prevalence of toxoplasmosis was greater in the lowlands as compared with the highland districts of Middle Java, Indonesia. One possible explanation could be the contamination of water sources from drifting soil, in the event of flooding or heavy rain, transporting oocysts from high to low ground, thus increasing the prevalence of oocysts in low-lying areas (Iskandar 1999).

Wonosobo and Banjarnegara had no cases of toxoplasmosis in this study. These are highland districts, with mountainous terrain, which may also affect the distribution of cats. As the cat is the definitive host of *T. gondii*, (Tavassoli et al. 2013), fewer cats in the environment will reduce the risk of transmission to the environment and livestock, thereby reducing the prevalence of toxoplasmosis in the local population.

Daily contact with raw meat was also identified as a risk factor for toxoplasmosis. Toxoplasmosis can occur during the transmission of *T. gondii* via the blood through a wound (CCOHS 2011). Daily contact with raw meat from infected animals increases the possibility of contact with tissue cysts, particularly if no protective equipment, such as gloves, is worn. Additionally, tissue cysts may be ingested during hand-to-mouth contact after handling undercooked meat, or from using knives, utensils, or cutting boards contaminated by raw meat (CDC 2004). This will also increase the risk of transmission in people who have regular contact with raw meat.

Consumption of unfiltered water was also identified as a risk factor for toxoplasmosis. Respondents who used non-filtered water were at increased risk of toxoplasmosis compared with those using a filtered water source. Consumption of unfiltered water was associated with water sources such as rivers, wells, and reservoirs. Oocysts can be transported via rivers and water during periods of heavy rain. Water is not just used for human consumption, but also for washing vegetables and as drinking water for livestock. The majority of respondents in Cilacap used filtered water. Cilacap is near the coast and because of the potential seawater contamination of water sources in this district, the use of filtered water is more widespread compared with other districts in Middle Java. This may be one factor leading to minimal cases of toxoplasmosis in several locations in Cilacap. Water is filtered using chlorine to reduce bacteria and parasites. Oocysts cannot survive for long in cold water conditions or in warm water with high levels of chlorination, UV, and ozone treatment (Wainwraight et al. 2007; Gangneux and Marie 2012).

This study identified a relationship between the population of cats and toxoplasmosis cases; people living in districts with high density of cats, Purworejo and Kebumen, were at increased risk of toxoplasmosis compared with those living in districts with low density of cats. Middle Java in general has a relatively small cat population, which varies in each district. A small number of cats do not necessarily indicate there is no risk of infection in that location. Cat feces take a long time to decay (Gangneux and Marie 2012), and during that time thousands of oocysts can develop (Ishaku et al. 2009).

In the surveyed population, men were at increased risk of infection compared with women. This could be associated with the differing roles of men and women in Middle Java; male-dominated professions, such as farming and meat production, will increase the risk of contact with contaminated meat, as well as increasing the risk of environmental exposure through spending long periods of time outdoors or in high-risk areas.

## Conclusion

This study highlighted the high prevalence of toxoplasmosis in Middle Java, and identified some important environmental, ecological, and demographic risk factors. Low elevation, contact with raw meat, unfiltered water sources, and the high density of cats are supposed to be risk factors of toxoplasmosis in Middle Java. Modifiable risk factors, such as unfiltered water sources and direct contact with raw meat, are relatively straightforward to address by encouraging people to use filtered water and wear protective clothing, such as gloves, when handling raw meat. The oocyst from infected cats will be transmitted to the environment through water shed and flooding and distributed from high land to low-lying areas.

## References

[CR1] Artama WT, Iskandar T, Widayanti R, Haryanto A (2007) Kloning, ekspresi gen penyandi protein excretory secretory antigen (esas) dan aplikasi protein rekombinan untuk menentukan prevalensi toksoplasmosis pada sapi, kambing dan domba di indonesia. http://www.litbang.pertanian.go.id/ks/one/406/file/kloning-ekspresi-gen-penya.pdf. Accessed Aug 25, 2015

[CR2] Behnke MS, Tiange PZ, Jitender PD, David S (2014). *Toxoplasma gondii* merozoit gene expression analysis with comparison to the life cycle discloses a unique expression state during enteric development. Biomed Central Genomics.

[CR3] Canadian Centre for Occupational Health and Safety (2011) Toxoplasmosis. https://www.ccohs.ca/oshanswers/diseases/toxoplasmosis.html. Accessed Aug 30, 2016

[CR4] Centers for Disease Control and Prevention (2004) Toxoplasmosis. http://www.cdc.gov/parasites/toxoplasmosis/epi.html. Accessed Aug 25, 2015

[CR5] Charan J, Tamoghna B (2013). How to calculate sample size for different study designs in medical research?. Indian Journal of Psychological Medicine.

[CR6] Davis JT (2012). The application of GIS and spatiotemporal analyses to investigations of unusual marine mammal strandings and mortality events. Marine Mammal Science.

[CR7] ESRI (2001) Spatial analysis: Advanced GIS spatial analysis using raster and vector data. https://www.esri.com/library/whitepapers/pdfs/arcgisspatialanalyst.pdf. Accessed March 24, 2014

[CR8] Fromont EG, Riche B, Rabilloud M (2009). *Toxoplasma* seroprevalence in a rural population in France: Detection of a household effect. BMC Infectious Diseases.

[CR9] Fusco G, Rinaldi L, Guarino A, Proroga YTR, Pesce A, Giuseppina DM, Cringoli G (2007). *Toxoplasma gondii* in sheep from Campania region (Italy). Veterinary Parasitology.

[CR10] Gangneux RF, Marie LD (2012). Epidemiology of and diagnostic strategies for toxoplasmosis. Clinical Microbiology Reviews.

[CR11] Gilot-Fromont E, Maud L, Marie-Laure D, Céline R, Dominique A, Eve A, Aurélien M, Cécile G, Isabelle V (2012) The life cycle of *Toxoplasma gondii* in the natural environment. http://www.intechopen.com/books/toxoplasmosis-recent-advances/the-life-cycle-of-toxoplasma-gondii-in-the-natural-environment. Accessed Aug 30, 2016

[CR12] Glasner PD, Silveira C, Kruszon-Moran D, Martins MC, Burnier JM, Silveira S, Camargo ME, Nussenblatt RB, Kaslow RA, Belfort JR (1992). An unusually high prevalence of ocular toxoplasmosis in Southern Brazil. American Journal of Ophthalmology.

[CR13] Hung CLH, So MK, Connell DW, Fung CN, Lam MHW, Nicholson S, Richardson BJ, Lam PKS (2004). A preliminary risk assessment of trace elements accumulated in fish to the Indo-Pacific humpback dolphin (*Sousa chinensis*) in the northwestern waters of Hong Kong. Chemosphere.

[CR14] Hung CLH, Lau RK, Lam JC (2007). Risk assessment of trace elements in the stomach contents of Indo-Pacific humpback dolphins and finless porpoises in Hong Kong waters. Chemosphere.

[CR15] Ishaku BS, Ajogi I, Umoh JU, Lawal I, Randawa AJ (2009). Seroprevalence and risk factors for *Toxoplasma gondii* infection among antenatal women in Zaria, Nigeria. Research Journal of Medicine & Medical Science.

[CR16] Iskandar T (1999). Tinjauan tentang toxoplasmosis pada hewan dan manusia. Wartazoa.

[CR17] João MF, Justine RS, Kevin LW (2011). Toxoplasmosis: A global threat. Journal Global infectious diseases.

[CR18] Jores JAE, Derocher AE, Staubach C, Aschfalk A (2008). Occurrence and prevalence of *Clostridium perfringens* in polar bears from Svalbard, Norway. Journal of Wildlife Diseases.

[CR19] Kamani J, Mani AU, Egwu GO, Kumshe HA (2009). Seroprevalence of human infection with *Toxoplasma gondii* and the associated risk factors, in Maiduguri, Borno state, Nigeria. Annals of Tropical Medicine Parasitology.

[CR20] Katlyn EW, Melissa AM, Bradd CB, Ian AG, Ann CM, Tim E, Andrea EP, Tin T, Manuel LS, Patricia AC (2007). Chemical inactivation of *Toxopasma gondii* oocysts in water. Journal of Parasitology.

[CR21] Miller MA, Gardner IA, Kreuder C, Paradies DM, Worcester KR, Jessup DA, Dodd E, Harris MD, Ames JA, Packham AE, Conrad PA (2002). Coastal freshwater runoff is a risk factor for *Toxoplasma gondii* infection of southern sea otters (Enhydra lutris nereis). International Journal for Parasitology.

[CR22] Miller MA, Grigg ME, Kreuder C, James ER, Melli AC, Crosbie PR, Jessup DA, Boothroyd JC, Brownstein D, Conrad PA (2004). An unusual genotype of *Toxoplasma gondii* is common in California sea otters (Enhydra lutris nereis) and is a cause of mortality. International Journal for Parasitology.

[CR23] Lee W, Hye-Jin A, Je-Hyun B, Chong-Heon L, Yeon GY, Ho-Woo N (2014). Comprehensive proteome analysis of the excretory/secretory proteins of *Toxoplasma gondii*. Bulletin of the Korean Chemical Society.

[CR24] Rahbari AH, Kesharvaz H, Schojae S, Mohebale M, Rezaeian M (2012). IgG avidity ELISA test for diagnosis of acute toxoplasmosis in humans. Korean Journal Parasitology.

[CR25] Rosen B (1997). On sampling with probability proportional to size. Journal of Statistical Planning and Inference.

[CR26] Shin DW, Cha DY, Hua QJ, Cha GH, Lee YH (2009). Seroprevalence of *Toxoplasma gondii* infection and characteristics of seropositive patients in general hospitals in Daejeon, Korea. Korean Journal Parasitology.

[CR27] Simon A, Michel BP, Alain NR, Nicholas HO (2013). Fate and transport of *Toxoplasma gondii* oocysts in seasonally snow covered watersheds: A conceptual framework from a melting snowpack to the Canadian Arctic Coasts. International Journal Environmental Research Public Health.

[CR28] Sulistyaningsih E, Moeljopawiro S, Subandono J, Artama WT (2005). Cloning of cDNA encoding GRA1 protein of tachyzoite *Toxoplasma gondii* local isolate. Indonesia Journal of Biotechnology.

[CR29] Suyatno (2013) Menghitung besar sampel penelitian kesehatan masyarakat. Fakultas Kesehatan Masyarakat UNDIP Semarang. http://www.slideshare.net/tobrono/menghitungbesarsampelpenelitian. Accessed March 24, 2014

[CR30] Swai ES, Schoonman L (2009). Seroprevalence of *Toxoplasma gondii* infection amongst residents of Tanga district in north-east Tanzania. Tanzan Journal Health Research.

[CR31] Tamavo S (2014). Evolutionary repurposing of endosomal systems for apical organelle biogenesis in *Toxoplasma gondii*. International Journal for Parasitology.

[CR32] Tavassoli M, Ghorbanzadehghan M, Esmaellnejad B (2013). Detection of *Toxoplasma gondii* in sheep and goats blood samples by PCR-RFLP in Urmia. Veterinary Research Forum.

[CR33] Tenter AM, Heckeroth AR, Weiss LM (2000). *Toxoplasma gondii*: from animals to humans. International Journal Parasitology.

[CR34] Uttah E, Emmanuel O, Christiana O (2013). Toxoplasmosis: A global infection, so widespread, so neglected. International Journal of Scientific and Research Publication.

[CR35] United Nations (2005) Designing household survey samples: practical guidelines. http://unstats.un.org/unsd/demographic/sources/surveys/Handbook23June05.pdf. Accessed March 24, 2014

[CR36] Wang Y, Yin H (2014). Research progress on surface antigen 1 (SAG1) of *Toxoplasma gondii*. Parasites and Vectors.

[CR37] Webster JP, Maya K, Greg CB, Glenn AM (2013). *Toxoplasma gondii* infection, from predation to schizophrenia: Can animal behaviour help us understand human behaviour?. Journal of Experimental Biology.

[CR38] Weis LM, Kami K (2014). *Toxoplasma gondii* The model of Apicomplexan: Perspective Methods.

[CR39] West MD (2008) Use of the Chi-square statistic. http://ocw.jhsph.edu/courses/fundepiii/pdfs/lecture17.pdf. Accessed March 24, 2014

[CR40] Wing EJ (2016). Toxoplasmosis: Cats have it, humans get It, but how much disease does it cause?. Clinical Infectious Diseases.

[CR41] Xiao Y, Yin J, Jiang N, Xiang M, Hao L, Lu H (2010). Seroepidemiology of human *Toxoplasma gondii* infection in China. BMC Infectious Diseases.

